# Cambrian problematica and the diversification of deuterostomes

**DOI:** 10.1186/1741-7007-10-79

**Published:** 2012-10-02

**Authors:** Andrew B Smith

**Affiliations:** 1 Department of Earth Sciences, The Natural History Museum, Cromwell Road, London SW7 5BD, UK

## Abstract

Vetulicolians are an enigmatic group of Cambrian organisms that have been affiliated at various times with arthropods, lobopodians, kinorhynchs and deuterostomes. New evidence on the structure of the lateral pores of vetulicolians published in *BMC Biology *strengthens the view that they may be total group deuterostomes, but unfortunately sheds no new light on early deuterostome evolution.

See research article http://www.biomedcentral.com/1741-7007/10/81

## 

Molecular data now provide a robust phylogenetic framework that reveals how morphologically disparate groups of organisms are related and establishes the relative order in which branches arose. This is vital information for understanding the evolution of life, yet it provides little constraint when reconstructing the ancestral morphologies that once must have existed or the detailed pathways along which evolution proceeded in the process of diversification. Here paleontology comes into its own, as fossils capture morphologies of organisms that belonged to the common stem group of living sister taxa but which have since been lost through extinction (for terminology, see Figure 1). Who could seriously have predicted the existence of stem group birds such as *Tyrannosaurus *from just studying the morphological diversity of modern birds? One of the undoubted strengths of the fossil record is that it allows paleontologists to recognize and order the steps involved in arriving at crown-group body plans [1]. So long as fossils can be placed with confidence within this evolutionary framework they have an important role to play. But sometimes the morphology of fossil groups is so bizarre or poorly understood that even to place them at the highest of taxonomic levels is difficult and controversial. Such is the case with the vetulicolians, a clade of organisms known from a handful of Cambrian deposits where soft-tissue preservation has occurred. Vetulicolians have the outward appearance of arthropods, with a posterior jointed appendage and a valved body, both of which are cuticularized to a certain degree. However, there are no signs of legs or antennae and the body bears a series of circular structures, five in number, that are interpreted by some as pores, by others as digestive glands. In the face of such a peculiar mixture of traits vetulicolians have, at different times, been affiliated with lobopodians, arthropods, kinorhynchs and deuterostomes. However, convincing synapomorphies (derived traits shared by the descendents from a common ancestor) supporting these phylogenetic placements have been lacking, leaving the question of the affinities of vetulicolians unresolved [2]. In the paper by Ou *et al. *[3], careful dissection and preparation of new vetulicolian specimens has revealed for the first time the fine structure of their lateral pores, and led the authors to conclude that vetulicolians are the most primitive known deuterostomes.

**Figure 1:**
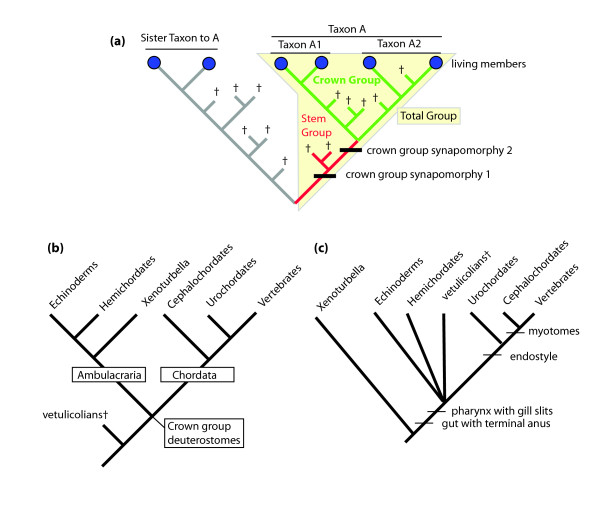
**Crown group deuterostome relationships**. **(a) **Diagram showing the relationships between crown group (green), stem group (red) and total group (yellow). A1 and A2 are sister taxa within the crown group; † = extinct taxon. Note that members of the stem group must have at least one but not all of the synapomorphies defining the crown group to be recognized as such. **(b) **Deuterostome relationships as deduced from molecular data [10] with vetulicolian placement following Ou *et al. *[3]. **(c) **Deuterostome relationships that would be inferred (wrongly) from adult morphology alone. The position of vetulicolians assumes their pore structures are indeed pharyngeal gill slits.

Ou *et al. *[3] set out the clearest case yet for why the five lateral structures on the valves of vetulicolians are pores connecting the interior of the body to the exterior. The arrangement of serially repeated pores piercing the body wall behind an oral opening is precisely how pharyngeal gill slits are arranged in deuterostomes, and is a diagnostic feature of that clade. The fossil material is not easy to work with, and their interpretation hinges on compressed fossils, with differential darkening of the sediment all there is to go on. But the evidence is there for all to see and a compelling case is developed. So far so good, and it is a major advance to be able to document the structure of these pores in such detail. However, this is not where the paper stops. Ou *et al. *[3] go on to make two further deductions: (i) that the pores formed an integral part of an active pumping system for feeding that is analogous but not homologous to that in tunicates; and (ii) that vetulicolians are stem group deuterostomes and thus reveal the body plan organization of deuterostomes prior to their split into echinoderms plus hemichordates and tunicates, chordates and cephalochordates. Here the reader needs to be more wary.

While the scenario woven concerning the function and dynamics of the pores in feeding is internally consistent with the observed morphology, this does not necessarily make it the only or even the most likely interpretation. Comparative anatomy of living deuterostomes suggests that pharyngeal gill slits first evolved to cope with excess water intake associated with ciliary-driven feeding [4]. The proposed feeding mechanism of active pumping in vetulicolians is distinctly different from that in all other deuterostomes except tunicates and hinges partially on observation that there are fine tissue strands internally rung longitudinally and vertically that are interpreted as muscle fibers, but which could just as easily be pharyngeal mesh. Unfortunately there is no current way of determining the kind of soft tissue from the nature of the faint dark stains in the rock. While Ou *et al*.'s interpretation is plausible and invites comparison with tunicates, in the end it is simply *ad hoc *and stretches the meager evidence. Speculation of course has its place, but, as paleontologists before have found to their cost, detailed scenarios can easily build to become a house of cards.

The second deduction made, that vetulicolians are stem group deuterostomes, is an even bolder claim as it carries far-reaching implications. If Ou *et al. *[3] are correct, then vetulicolians provide key evidence for the body plan of deuterostomes prior to their divergence into the modern phyla (Figure 1b). But can we be sure that vetulicolians are stem group deuterostomes? Here we run into the problem of the phylogenetic resolution that can be achieved by reference to adult morphology alone. Until very recently relationships amongst the five major deuterostome phyla had proved impossible to resolve because adult morphological traits informative about basal deuterostome relationships simply do not exist, and even those from embryology are few [5]. Indeed, it was only with the advent of molecular data that satisfactory phylogenetic resolution of the deuterostome phyla was finally achieved. To be identifiable as a member of the stem group of deuterostomes a fossil would have to show some but not all of the crown group synapomorphies (Figure 1a). There remains a considerable degree of uncertainty about what characters the latest common ancestor to all deuterostomes would have displayed [5]. However, one generally accepted model is that the latest common ancestor of deuterostomes was a worm-like creature with pharyngeal gill slits, a terminal anus, a simple nerve plexus without regionalization, and well-developed circular and longitudinal muscles [6]. Vetulicolians apparently have pharyngeal gill slits, a terminal anus and possibly longitudinal and circular body wall musculature but other key aspects of their anatomy and embryology remain unknown. So the best we can say is that they belong to the total group Deuterostomia and lack clear synapomorphies with any crown phylum. An inability to find derived characters shared with any crown group deuterostome is insufficient argument to place them as stem group deuterostomes - it is even possible that they could be an early, extinct side-branch of one of the major deuterostome phyla. When first described [7], a possible endostyle was identified in vetulicolians (though the evidence for this is tenuous at best), which would place them on the chordate branch of deuterostomes. As morphological data do not support the molecular tree (Figure 1c), resolving the position of primitive fossil deuterostomes is fraught with difficulties. For example, the earliest echinoderms are believed to be bilaterally symmetrical with pharyngeal filtration feeding and gill slits [8], but we stand no chance of recognizing them as such until the first crown group synapomorphy - their calcitic skeleton of stereom - had evolved. Remember that divergence of crown group deuterostomes occurred in the late pre-Cambrian according to the best molecular clock estimates [9] and, by the time vetulicolians appear in the fossil record, diversification within crown group deuterostome phyla was already well underway.

Finally, it is important to bear in mind that fossils can only be interpreted in the light of our understanding of the extant biota. Fossils cannot tell us that pharyngeal gill slits are a key deuterostome feature, as the presence of pharyngeal gill slits can only be inferred based on the superficial similarity of appearance, not on observed function. It was optimizing morphological traits of extant organisms onto molecular phylogenies, not fossils, that convincingly demonstrated pharyngeal gill slits to be a synapomorphy for all deuterostomes and thus present in their latest common ancestor [4]. So while this paper is an important step forward in understanding and clarifying the morphology of vetulicolians and makes their position as deuterostomes more likely, the idea that they inform us about the stem group of deuterostomes far oversteps what the data to hand allow. Given that it is currently impossible to define a stem group deuterostome based on adult morphological traits, vetulicolians, if indeed they have pharyngeal gill slits, must remain enigmatic total group deuterostomes.
